# COVID-19 multidisciplinary high dependency unit: the Milan model

**DOI:** 10.1186/s12931-020-01516-8

**Published:** 2020-10-09

**Authors:** Stefano Aliberti, Francesco Amati, Maria Pappalettera, Marta Di Pasquale, Alice D’Adda, Marco Mantero, Andrea Gramegna, Edoardo Simonetta, Anna Maria Oneta, Emilia Privitera, Andrea Gori, Giorgio Bozzi, Flora Peyvandi, Francesca Minoia, Giovanni Filocamo, Chiara Abbruzzese, Marco Vicenzi, Paola Tagliabue, Salvatore Alongi, Francesco Blasi

**Affiliations:** 1 IRCCS Fondazione Ca’ Granda Ospedale Maggiore Policlinico, Respiratory Unit and Cystic Fibrosis Adult Center, Via Francesco Sforza 35, 20122 Milan, Italy; 2 University of Milan, Department of Pathophysiology and Transplantation, Milan, Italy; 3 Infectious Disease Unit, Department of Internal Medicine, Fondazione IRCCS Ca’ Granda, Ospedale Maggiore Policlinico, Milan, Italy; 4 Fondazione IRCCS Ca’ Granda Ospedale Maggiore Policlinico, Angelo Bianchi Bonomi Hemophilia and Thrombosis Center, Milan, Italy; 5 Pediatric Rheumatology, Medium Intensity Care Unit, Fondazione IRCCS Ca’ Granda Ospedale Maggiore Policlinico, Milan, Italy; 6 Departement of Anesthesiology, Intensive Care and Emergency, Fondazione IRCCS Cà Granda Ospedale Maggiore Policlinico, Milan, Italy; 7 Cardiovascular Disease Unit, Internal Medicine Department, Fondazione IRCCS Ca’ Granda Ospedale Maggiore Policlinico, Milan, Italy; 8 Dyspnea Lab, Department of Clinical Sciences and Community Health, University of Milan, Milan, Italy

**Keywords:** COVID-19, High dependency unit, Multidisciplinary approach, Management

## Abstract

COVID-19 is a complex and heterogeneous disease. The pathogenesis and the complications of the disease are not fully elucidated, and increasing evidence shows that SARS-CoV-2 causes a systemic inflammatory disease rather than a pulmonary disease. The management of hospitalized patients in COVID-19 dedicated units is advisable for segregation purpose as well as for infection control. In this article we present the standard operating procedures of our COVID-19 high dependency unit of the Policlinico Hospital, in Milan. Our high dependency unit is based on a multidisciplinary approach. We think that the multidisciplinary involvement of several figures can better identify treatable traits of COVID-19 disease, early identify patients who can quickly deteriorate, particularly patients with multiple comorbidities, and better manage complications related to off-label treatments. Although no generalizable to other hospitals and different healthcare settings, we think that our experience and our point of view can be helpful for countries and hospitals that are now starting to face the COVID-19 outbreak.

## Background

On January 30, 2020, the World Health Organization (WHO) designated an outbreak of a novel coronavirus not seen before in humans to be a “public health emergency of international concern” [1]. On March 11, 2020, WHO declared the coronavirus disease 2019 (COVID-19) outbreak a global pandemic [2, 3]. In March 2020, Italy became the epicenter for COVID-19 in Europe, and the second most affected country after China worldwide [4]. Up to 20% of suspected and confirmed patients with Severe Acute Respiratory Syndrome coronavirus 2 (SARS-CoV-2) infection develop severe hypoxemia and require some forms of ventilatory support, such as high-flow nasal cannula (HFNC), non-invasive (NIV) or invasive mechanical ventilation (IMV). Furthermore, other important challenges have been identified soon after the disease development, including cardiac, renal, neurological and thromboembolic complications [5–9]. A rapid widespread of units dedicated to COVID-19 patients has characterized the public health scenario worldwide, including Italy and its more affected region, Lombardy [10]. The high number of people who got infected by SARS-CoV-2 and who developed acute respiratory failure (ARF) rapidly overcame the number of intensive care unit (ICU) beds in Lombardia, and this led several high dependency units (HDU) to be converted in specific COVID-19 HDU. The process of developing a COVID-19 HDU, along with its standard operating procedures (SOPs), could be useful for those who are preparing themselves for the COVID-19 epidemic or to implement already existing COVID-19 HDU. The aim of this article is to present the organization of the COVID-19 HDU of the Policlinico Hospital in Milan, Italy. Although we are aware that the procedures adopted in our hospital cannot be entirely generalized in other hospitals and healthcare systems, we think that the information of the paper should be shared with the clinical community in order to help colleagues to develop or implement a multidisciplinary COVID-19 HDU.

## The multidisciplinary COVID-19 HDU

The Fondazione IRCCS Ca′ Granda Ospedale Maggiore Policlinico is the oldest hospital in Milan, located in the city centre, and organized in different pavilions (Fig. 1, online supplements). The pavilion-based structure has clear advantages for the management of infectious diseases, including COVID-19, since it allows microbiological segregation. During the COVID-19 outbreak in Milan, four different pavilions of the Policlinico Hospital have been entirely allocated for the management of COVID-19 patients, including one emergency room (ER), four ICUs, two HDUs and four low-dependency units. Our respiratory HDU was converted to COVID-19 HDU and the entire pulmonary team (pulmunologists, nurses, respiratory physicians) was involved in the care of COVID-19 patients. The same process of segregation has been adopted by other services (e.g radiology). The number of HDU and ICU COVID-19 beds available in our hospital have been about 3 times the number of HDU and ICU beds available before the start of the pandemic. Eligible COVID-19 patients for the 41-bed COVID-19 HDU have been discussed on a case-by-case basis to identify the best suitable setting, according to the following criteria: 1) the severity of ARF evaluated through the PaO_2_/FiO_2_ ratio; 2) The presence of immunosuppression; and 3) the presence of a colonization/infection with a multi-drug resistant (MDR) bacteria. Key information to triage COVID-19 patients who are about to be admitted to the COVID-19 HDU are: 1) The last PaO_2_/FiO_2_ ratio and type of respiratory support (e.g. HFNC, helmet Continuous Positive Airway Pressure (CPAP), and NIV); 2) The presence of an infection/colonization with a MDR bacteria; 3) The state of consciousness/anxiety/sedation; 4) The indication concerning the Do Not Resuscitate (DNR)/Do Not Intubate (DNI) *status*; 5) Patient’s performance *status*; 6) The presence of signs of sepsis; 7) The severity of involvement of other organs (heart, kidney, liver, central nervous system). The COVID-19 HDU do not only admits patients coming to the ER but also acts as a catchment area for other COVID-19 units (including ICU) of the Policlinico hospital and other hospitals in Lombardy.

We involved other healthcare professionals in our multidisciplinary team. Cardiologists, infectious diseases specialists, rheumatologists, intensivists and respiratory physiotherapists are fundamental components of the COVID-19 HDU, working together in a multidisciplinary team, see Table 1. The multidisciplinary approach to COVID-19 has a crucial role for several reasons. Firstly, the pathogenesis and the complications of the disease are not fully elucidated, and increasing evidence shows that SARS-CoV-2 causes a systemic inflammatory disease rather than a pulmonary disease [5–9, 11, 12]. Secondly, no licensed drugs exist for COVID-19, and the majority of treatments are off-label or within clinical trials [13–16]. Thirdly, management of complications and adverse events of drugs require a multidisciplinary approach, especially in patients with multiple comorbidities [9]. Finally, a daily clinical discussion with other COVID-19 units is crucial to quickly identify patients who would benefit from invasive support or transfer to a low-dependency unit.

**Table 1:** Healthcare professionals involved in the multidisciplinary team

**Respiratory physician**	• Initial evaluation of patient • Choice of respiratory support • Evaluation and placement of central venous catheter and/or arterial catheter • Identify signs of sepsis or multi-organ failure • Setting of sedative therapy, nutritional therapy, anti-thrombotic prophylaxis, hydration, antiviral and antibiotic therapy
**Fellow**	• Initial evaluation of patient • Placement of arterial catheter • Blood and microbiological tests request • Arterial blood gas test • Pneumonia follow-up with lung ultrasound
**Nurse**	• Preparation of medical devices to support respiratory insufficiency • EKG • Placement of peripheral venous catheter • Placement of bladder catheter • Collection of vital parameters • Therapies administration
**Respiratory physiotherapist**	• Evaluation with respiratory physician of ventilator/oxygen support • Early mobilization
**Cardiologist**	• Evaluation and placement of central venous catheter and/or arterial catheter • Identification and management of cardiac complications • Anti-hypertensive therapy • Inotropic support
**Infectious disease specialist**	• Identification of patients candidate to anti-viral or anti-inflammatory therapy • Choice of antiviral drugs • Choice of antibiotic therapy • Identification and treatment of sepsis • Super-infection identification and management
**Rheumatologist**	• Identification of patients candidate to the anti-inflammatory and specific anti-cytokine treatment • Definition of a tailored anti-inflammatory strategy according to the patient characteristics
**Intensivist**	• Multidisciplinary discussion to early identify patients candidate to intensive care management • DNI/DNR status

*Abbreviations*: *EKG* Elettrocardiogram, *DNI* Do not intubate, *DNR* Do not resuscitate

## Standard operating procedures of the COVID-19 HDU

### Initial assessment of patient

A minimum bundle of tests through a systematic approach is implemented on the COVID-19 HDU. Additional tests should be considered on a case-by-case basis according to clinical condition and specific comorbidities, see Table 2.

**Table 2:** Minimum bundle of tests that are performed on HDU admission

Minimum bundle of test on HDU admission	Additional test
**Vital parameters** (body temperature, blood pressure, respiratory rate, diuresis, ventilator/oxygen parameters) on admission and after 1, 3 and 6 h after admission.	**Thorax CT** based on clinical status and risk of superinfection/pulmonary complications (contrast medium should be strongly considered if clinical suspicion of pulmonary embolism is present).
**BGA** on admission and after 1 and 3 h after admission	**Throat cultures for MRSA** and **rectal swab for multi-resistant pathogens** in case the patient is transferred from an intensive care unit or any other unit
**Blood tests**: blood cell count, CRP, PCT, transaminase, creatine phosphokinase (CPK), lactate dehydrogenase (LDH), total bilirubin, gamma-glutamyl transferase (GGT), alkaline phosphatase (ALP), creatinine, serum sodium, serum potassium, serum clorum, glycemia, albumin, prothrombin time (PT), activated partial thromboplastin time (aPTT or APTT), D-dimer, ferritin, fibrinogen, triglycerides, proBNP, troponin T, IL-6)	**Echocardiography i**n case of clinical suspicion of myocardial/pericardial involvement or heart failure
**Microbiological tests**: *L. pneumophila* and *S. pneumoniae* urinary antigen; blood cultures (preferably during fever)	
**EKG**	
**Chest-X ray** if not performed in the last 3 days	
**Lung ultrasound**	
**Lower extremity venous ultrasound**	
**Inferior vena cava ultrasound**	

*Abbreviations*: *HDU* High dependency unit, *CT* Computed tomography, *BGA* Blood gas analysis, *MRSA* Methicillin-resistant *Staphylococcus aureus, EKG* Elettrocardiogram

### Respiratory support

- ▪ COVID-19 patients are stratified according to severity and type of ARF on HDU admission with the aim to set respiratory support on a single individual basis [17], see Table 3.

**Table 3:** Proposed respiratory support based on the severity of acute respiratory failure

Acute Respiratory failure	Alternative
▪ P/F ratio > 300 and respiratory rate (RR) < 30	▪ Low-flow nasal cannula oxygen or Venturi Mask or Reservoir Mask set with the aim of target SpO2 92–96%
	▪ HFNC 40 L/min and FiO2 set with the aim of target SpO2 92–96%
▪ P/F ratio 100–300 and RR < 30	▪ Helmet CPAP with PEEP 5 or 7.5 cmH2O and FiO2 set with the aim of target SpO2 92–96%
▪ P/F ratio < 100 and RR < 30	▪ Helmet CPAP with PEEP 5 or 7.5 cmH2O and FiO2 set with the aim of target SpO2 92–96%
▪ P/F ratio < 100 and RR ≥ 30 and/or respiratory distress	▪ NIV (Also to consider in case of: CPAP failure, hyper-capnia). NIV starting parameters: PEEP 12–16 cmH20 PS set with the aim of Vt 4–6 ml/kg and FiO2 set with the aim of target SpO2 90–95%

*Abbreviations*: *P/F ratio* arterial pO2 divided by the fraction (percent) of inspired oxygen, *HFNC* High-flow nasal cannula, *CPAP* Continuous positive airway pressure, *FiO2* fraction (percent) of inspired oxygen, *NIV* Non-invasive ventilation, *PEEP* Positive end-expiratory pressure

COVID-19 is a very heterogeneous disease and the type and severity of ARF depends on the interaction among multiple factors including the time from symptoms onset and admission to HDU, the severity of the infection, the host response, physiological reserve and comorbidities, and the ventilatory responsiveness of the patient to hypoxemia [9, 18, 19]. A systematic review and meta-analysis of 25 randomized control trials (RCTs) showed that a liberal oxygen strategy (SpO2 targets higher of 96%) is associated with increased risk of hospital mortality in acutely ill patients [20]. In the HDU FiO2 is settled with the aim of target SpO2 of 92–96%. Despite international guidelines recommending only cautious trials of NIV in immunocompetent patients with ARF due to community-acquired pneumonia (CAP), RCTs showed that the possible application of Positive End-expiratory Pressure (PEEP) in CAP patients is able to recruit alveoli leading to a rapid improvement in oxygenation [21–23]. However, NIV and CPAP should not delay endo-tracheal intubation in patients who could benefit of invasive ventilation [24]. In particular, intubation should be prioritized in patients treated with CPAP or NIV presenting with clinical signs of excessive inspiratory efforts, to avoid excessive intrathoracic negative pressures and self-inflicted lung injury [25]. Levels of PEEP and pressure support during CPAP or NIV should be individualized to obtain the lowest level of support able to oxygenate the patient without increase the risk of both lung and cardiovascular side effects. A particular consideration shall be given to high PEEP pressures considering the increase risk of pneumothorax/pneumomediastinum. Furthermore, high PEEP in a poorly recruitable lung tends to result in severe haemodynamic impairment and fluid retention [26]. This is the rationale for the implementation in the SOPs of the COVID-19 HDU of the “zero end-expiratory pressure (ZEEP)-PEEP test” to tailor PEEP level in each single patient [27]. Patient posture during NIV is crucial to optimize ventilation. In particular, slumped posture should be avoided and early mobilization for all patients is encouraged. Prone positioning or lateral position could be also considered in these patients according to imaging and clinical status [28, 29]. Preliminary evidences show improvements in oxygenation parameters with prone positioning in patients with COVID-19 receiving NIV or HFNC [30]. However, clinicians should be aware that prone positioning can be also harmful [31]. Indeed, prone positioning of patients with relatively high compliance results in a modest benefit at the price of a high demand for stressed human resources [32]. Close blood gas analysis (BGA) and clinical evaluation are performed after position changes to verify the advantages. Finally, all the medical devices for non-invasive respiratory support have a risk of droplet spreading [33, 34]. The risk of infection spread is higher with HFNC (a surgical mask should be put on by the patient) and lower with helmet CPAP [34]. This is one of the reasons why we decided to prefer helmet CPAP in the COVID-19 HDU. CPAP and NIV should be equipped with a double filter (in and out) to minimize the risk of droplet spreading. Heat and moisture exchanger (HME) filters should be positioned before the PEEP resistance and on inspiratory circuit of Helmet. However, the use of out-put filter positioned before the PEEP resistance can increase the pressure of 2.5–5 cmH20 (high variability of data) in the circuit. Aerosol and warm humidifiers are avoided in our HDU to prevent droplet diffusion.

### Antiviral therapy

- ▪ Remdesivir (clinical trial)

Despite several randomized trials being underway to evaluate the safety and efficacy of different antiviral agents, data supporting the use of any anti-viral agents in COVID-19 have been mostly extrapolated from case series or in vitro experiments [14–16, 35, 36]. The multidisciplinary team of the COVID-19 HDU discussed the pro and cons and the evidence of introducing an antiviral drug in patients with COVID-19 pneumonia and acute respiratory failure, and initially decided to consider the use of hydroxychloroquine for all patients in absence of contraindications [35–37]. In vitro, hydroxychloroquine hampers low pH-dependant steps of viral replication resulting in antiviral activity [38]. Retrospective and open-label non-randomized clinical trial in patients hospitalized with COVID-19 showed that hydroxychloroquine was associated with decreased mortality and viral load reduction [35, 36]. Before starting hydroxychloroquine treatment, an electrocardiogram (EKG) was performed given the risk of QT prolongation and interactions with other drugs was checked [39]. EKG was usually repeated every 48 h to assess the risk of QTc prolongation. Indeed, studies have highlighted the potential for toxicity of hydroxychloroquine, particularly QTc prolongation and risk of arrhythmias [40, 41]. However, accumulating data from trials and observational studies suggested that hydroxychloroquine do not provide a clinical benefit regards of mortality or leght of hospital stay for patients with COVID-19 [40–42]. According to this data the multidisciplinary team decided to not routinely use hydroxychloroquine in COVID-19 patients. After multidisciplinary discussion, the COVID-19 team decided again the use of lopinavir/ritonavir according to evidence showing no benefit of this treatment beyond standard of care [13]. Furthermore, lopinavir/ritonavir is associated with gastrointestinal adverse events leading to discontinuation of treatment in 14% of the patients within a clinical trial [13]. Remdesivir, a broad-spectrum antiviral with potent in vitro activity against a range of RNA viruses, has shown in vivo activity against related coronavirus MERS-CoV and SARS-CoV-2 animal models [15, 16]. Furthermore, preliminary data from randomized trials suggest clinical benefit of remdesivir in hospitalized COVID-19 patients [14, 43]. In the COVID-19 HDU, remdesivir is used within a clinical trial programme [14].

### Anti-inflammatory therapy

- ▪ Methylprednisolone alone in patients with all the following criteria: CPAP / NIV support; procalcitonin < 1 μg/L; active inflammation (C-reactive Protein> upper limit of normal and/or fever without alternative origin); no clinical signs of sepsis. Methylprednisolone dosage is 80 mg/die intravenous (consider 120 mg/die in severe cases).
- ▪ Anakinra (anti IL-1) and methylprednisolone in patients with all the following criteria: CPAP / NIV support; ferritin > 1.000 ng/ml; procalcitonin (PCT) < 1 μg/L; no clinical signs of sepsis; no other biologics. Anakinra dosage is 200 mg subcutaneously every 8 h followed by 100 mg every 6 h according to inflammatory markers and ferritin, associated with methylprednisolone dosage is 80 mg/die intravenous (consider 120 mg/die in severe cases) [5, 44]. The most common Anakinra side effects are wheal at the injection site and neutropenia [45].
- ▪ Tocilizumab (anti IL-6) in patients with all the following criteria: IL6 > 5 x ULN; transaminase levels lower than × 5 upper limit of normal; neutrophils > 500 cell/mm3; platelets > 50,000 cell/mm3; no clinical signs of sepsis; no diverticulitis nor bowel perforation; no systemic or cutaneous infection; no other biologics. Tocilizumab dosage is (anti IL-6) 8 mg/kg (weight > 30 kg) or 12 mg/kg (weight < 30 kg). Adverse events are: upper respiratory tract skin and soft tissue infections; headache; transient decreases in neutrophil count; abnormal liver function tests; nasopharyngitis; and diarrhea [46, 47].

Accumulating evidence suggests that a subgroup of patients with severe COVID-19 have laboratory and clinical evidence of an exuberant inflammatory response. This constellation of features is reminiscent of a family of syndromes broadly gathered under the umbrella of cytokine storm syndrome, in which hyperinflammation and multi-organ disease arise through excessive cytokine release from uncontrolled immune activation, with persistent fevers, elevated inflammatory markers, and elevated pro-inflammatory cytokines [11, 12]. These “endotypes” have been associated with critical and fatal illnesses [11]. Therefore, identification of biomarkers such as ferritin or IL-6, and treatment of these biomarkers stimulating hyper-inflammation using approved or under-evaluation therapies is of paramount importance to reduce the rising mortality. Indeed, predictors of mortality from a multicentre retrospective study of confirmed COVID-19 cases in China included elevated ferritin and IL-6, suggesting that mortality might be due to virally driven hyper-inflammation [48]. Therapeutic options to target specific pathways leading to hyper-inflammation include selective cytokine blockade agents such as anakinra or tocilizumab [5, 44, 49]. However, no randomized control trial has been published to support the use of these drugs in COVID-19 disease. The use of these drugs has been extrapolated from case series, observational studies or is based on trials on other diseases that leads to viral-driven hyper-inflammation [12, 44, 49–53]. Finally, the use of steroid therapy to control the hyper-inflammation is largely debated. The WHO, the Centers for Disease Control (CDC) and the Infectious Diseases Society of America (IDSA) does not recommend glucocorticoids in patients with COVID-19 pneumonia because they have been associated with an increased risk for mortality in patients with influenza and delayed viral clearance in patients with Middle East respiratory syndrome coronavirus (MERS-CoV) infection [54–56]. However, a recent observational study from China suggested that the use of corticosteroids may reduce mortality in COVID-19 patients with Acute Respiratory Distress Syndrome (ARDS), so systemic corticosteroids could be considered in moderate to severe ARDS in our unit, as suggested by Surviving Sepsis Campaign [5, 57]. During the writing of the present manuscript, dexamethasone has been approved in the United Kingdom for all hospitalized patients with COVID-19 requiring oxygen [58].

### Antibiotic coverage

- Antibiotic coverage is considered in case at least one of the following criteria is present: 1) WBC < 4.000 o > 10.000 (and neutrophilia); 2) PCT > 1 μg/L (or PCT > 0.5 μg/L on two consecutive determination); 3) Clinical-radiological signs of bacterial infection. Antibiotic selection follows the latest IDSA/ATS CAP Guidelines [21].

Inappropriate antibiotic use may reduce their availability and broad-spectrum antibiotics in particular may lead to *Clostridium difficile* infection and antimicrobial resistance [21, 22]. For these reasons, we consider antibiotic therapy only in case of criteria suggesting risk of co-infection or super-imposed infection with bacteria. Broad spectrum antibiotic coverage is based on risk factors for *Methicillin-Resistant Staphylococcus aureus* (MRSA) or *Pseudomonas aeruginosa* [21]. Empiric broad spectrum antibiotic coverage is continued while obtaining definitive cultural data [21]. In patients tested positive for Influenza virus A or B, oseltamivir 75 mg 1 tablet every 12 h is initiated with the aim of reducing the risk of death [59, 60]. Finally, in case of treatment with a macrolides or respiratory fluoroquinolones, EKG is performed on daily basis especially if the patient is on hydroxychloroquine or other medications that prolong QT interval [39, 42, 61–65].

### Anti-thrombotic prophylaxis

- In patient with a low-risk of bleeding (normal clotting time with PT < 1.5, platelets > 100,000 mm^3^, no active bleeding, and no risk of trauma), an individualized prophylaxis with enoxaparin sodium is given subcutaneously (either 4000 IU every 24 h, or 100 IU/kg every 24 h, or 70 IU/kg every 24 h, according to disease severity and inflammatory and coagulative markers). In case of creatinine clearance < 30 ml/min, Heparin calcium at starting dose of 5000 Units/ 0.2 ml solution × 3 daily for subcutaneous injection was given (aPTT target: 2.0). Patients on chronic therapy with direct anti FII, anti FX oral anticoagulants or warfarin have been switched to enoxaparin sodium 100 IU/kg × 2/die subcutaneously.
- In patients with a high-risk of bleeding or abnormal clotting time, a multidisciplinary discussion is organized with haematologists in order to individualize anti-thrombotic prophylaxis on patient’s characteristics.

COVID-19 patients have an increased risk of pulmonary embolism (PE) and deep venous thrombosis (DVT), in particular those who are critically ill [6–8, 66–69]. A recent study enrolling 184 critically ill patients who received at least standard dose thromboprophylaxis found 31% incidence of thrombotic complications [68]. However, the exact frequency of these complications is unknown, and a large data collection through the WHO’s COVID19 Clinical Network is ongoing. Reasons leading to PE and DVT occurrence in COVID-19 patients are unclear. Evidence suggests that elevated D-dimer is common in COVID-19 patients, especially in those at severe stage [7, 9]. It is well known that elevated D-dimer is associated with a higher risk of PE and DVT, as well as other conditions such as cancer, peripheral vascular disease, pregnancy, and inflammatory disease [70]. Inflammation is one of the key mechanisms in the worsening of respiratory conditions in COVID-19 [12, 71]. Autopsy results of multiple series of SARS patients showed that vascular thromboses were common in lung specimens, suggesting an underlying thrombophilia in the lungs [8]. The predominant coagulation abnormalities in patients with COVID-19 suggest a hypercoagulable state called “thromboinflammation” or COVID-19-associated coagulopathy by some experts [7]. It appears to be distinct from disseminated intravascular coagulation (DIC), though DIC has been reported in severely affected patients [7, 72]. Furthermore, reduced physical movements can result in higher risk of DVT in patients’ lower limbs. Finally, a pre-clinical study shows that heparin bind structural proteins of SARS-CoV-2 inducing conformational changes. These data support the repurposing of heparin and its derivatives as potentially anti SARS-CoV-2 agents. For all these reasons, anti-thrombotic prophylaxis should be encouraged in patients with COVID-19 pneumonia determining respiratory failure [68]. We favour pharmacologic prophylaxis of venous thromboembolism consistent with recommendations from expert societies [57]. However, the intensity of thromboprophylaxis, especially in severe and moderate COVID-19 patients, is an outstanding question. Our approach is to apply pharmacological thrombosis prophylaxis in severe and moderate COVID-19 patients towards high prophylactic doses, even in the absence of randomized control trial evidence. However, physicians should start by being vigilant for signs of thrombotic complications, and order appropriate diagnostic tests at a low threshold.

### Other therapies

- ▪ Other therapies are crucial to control signs and symptoms related to ARF and systemic inflammation induced by COVID-19 disease, see Table 4.

**Table 4:** Other therapies for patients with COVID-19 disease

**Antipyretic**	• Paracetamol 1 g intravenous/orally every 8 h (with the goal to keep fever under control in patients with respiratory insufficiency) for all patients with body temperature > 37 °C. • Alternative: ○ Diclofenac 75 mg intravenous in 24 h. ○ Metamizole 500 mg intravenous every 8 h.
**Systemic hypertension treatment**	• Patients with systemic hypertension already on medication: antihypertensive therapy should be continued regardless of pharmacologic (ACE-inhibitor, sartan, beta-blocker) [73]. Diuretics should be discontinued to avoid hypovolemic status. • Patients that develop systemic hypertension during the hospitalization: treatment options include potassium-spring diuretics (spironolactone 50 mg × 2/die or potassium canreonate intravenous with a minimum dose of 100 mg × 2/die) associated with ACE-inhibitors or sartans.
**Hydration**	• Hydration should be considered in all patients (especially patients with fever). • Before start of treatment with CPAP or NIV hydration should be provided in patients with signs of hypovolemia.
**Nutrition**	• In patients that are able to eat in HFNC or nasal cannulas: self-sufficient oral feeding • CPAP or NIV-dependent: nasal feeding tube should be placed to provide enteral feeding (e.g.: isosource protein 25 Kcal/kg) • In selected cases parenteral feeding (after positioning of central arterial access): ○ 1. BMI ≥ 20 provide at least 1080 kcal (speed:1,5 ml/kg/h) ○ 2. BMI < 20 provide at least 1540 kcal (speed:1,5 ml/kg/h)
**Sedation**	• Anxious state: Alprazolam (starting dose 0,25 mg × 2/die orally) • Psychomotor agitation, attempt to remove medical devices, tachypnoea: morphine bolus (2,5 mg i.v./s.c., max every 6 h) +/− Alprazolam (starting dose 0,25 mg × 2/die). At least 2 h between administration of alprazolam and morphine.
**End of life support**	• Starting dose: syringe pump with morphine 10 mg + midazolam 5 mg + haloperidol 5 mg + metoclopramide 10 mg • Dose should be modified according to clinical condition of the patient
**Gastric protection**	• Omeprazole 20 mg every 24 h orally/intravenous
**Home therapy that should not be discontinued during hospitalization**	- Levothyroxine - Beta-blockers and others essential cardiological therapies - Insulin in diabetic patients (oral antihyperglycemic should be discontinued in case of P/F ratio < 300 or acute kidney injury) - Corticosteroid therapy (*decalage* should be encouraged based on clinical condition of underlying condition)

*Abbreviations*: *HFNC* High-flow nasal cannula, *CPAP* Continuous positive airway pressure, *NIV* Non-invasive ventilation, *BMI* Body mass index

Antipyretic medication in critically COVID-19 patients may reduce the risk of haemodynamic instability and hypoxic tissue damage, in particular for elderly patients and/or those with comorbidities [73]. These patients are more vulnerable to elevated physiological demands during high-grade fever presenting an increased risk of dehydration or circulatory dysregulation [74]. For these reasons, as suggested by the Surviving Sepsis Campaign guidelines on management of critically ill patients with COVID-19, anti-piretic agents are used. Furthermore, fluid management, especially in-patients with severe febrile illness, is of paramount importance. Antihypertensive therapy is another controversial area. Systemic hypertension is the most common comorbidity in COVID-19, especially in severe cases [75]. Preliminary studies speculated that the SARS-CoV-2 might enter the human body via angiotensin-converting enzyme 2 (ACE2) on the surfaces of type II alveolar cells [76, 77]. Furthermore, earlier studies about SARS-CoV pathogenesis proved the binding of virus protein S to ACE2 receptor, is associated with ACE2 down-regulation, excessive production of angiotensin by ACE and less vasodilatory function of heptapeptide angiotensin [78]. The renin-angiotensin system (RAS) plays an important role in the occurrence and development of hypertension, and ACE inhibitors (ACEIs) and angiotensin receptor antagonists (ARBs) are the main antihypertensive drugs recommended by the current guidelines. Despite the possible up-regulation of ACE2 by RAS inhibition, there is currently no data proving a causal relationship between ACE2 activity and SARS-CoV-2 associated mortality. Thus, antihypertensive therapy is continued in COVID-19 patients hospitalized in the HDU regardless of pharmacologic.

### Clinical stability and clinical failure in COVID-19

The concept of clinical stability is widely adopted into clinical practice for CAP patients [21, 79–81]. A variety of criteria for determining clinical improvement have been developed and validated in clinical trials for patients with CAP, including resolution of vital sign abnormalities (heart rate, respiratory rate, blood pressure, oxygen saturation, and temperature), ability to eat, and normal mental status [80]. Failure to achieve clinical stability within 5 days is associated with higher mortality and worse clinical outcomes in CAP patients [79, 82]. Translating the idea of clinical stability from CAP to COVID-19 is difficult because of the characteristics of the virus, the absence of antiviral licensed therapy, and the dis-regulation of the immune system. All these reasons make the evolution of the disease unpredictable with the risk of sudden worsening of pneumonia. Identifing patients who are refractory to initial treatment in COVID-19 pneumonia is of paramount importance. However, no validated definition of clinical failure exists in COVID-19 pneumonia and the same limitations described for clinical stability also applies to clinical failure. Based on our experience, clinical failure could be defined when one of the following criteria is identified: persistence of fever (TC > 37.5 °C) and/or no improvement in inflammatory parameters levels; no improvement or worsening in P/F ratio; tachypnoea and respiratory distress (respiratory rate > 30 breaths/minute); refractory hypoxemia; multiorgan failure; sepsis; new onset of complications.

### Criteria to identify patients who are candidate to intubation and invasive support

Two meetings with critical care colleagues are organized every day to discuss patients who are eligible for intubation as well as those who have been transferred from ICU for weaning. All patients with at least one of the following criteria are evaluated for invasive respiratory support: 1) CPAP/NIV dependency for at least 24 h; 2) PaO_2_/FiO_2_ ratio < 150; 3) Respiratory rate > 30 and respiratory distress despite optimization of non-invasive respiratory support; 4) Hypercapnia and/or pH < 7.30; 5) Intolerance to respiratory support interface leading to discontinuation of respiratory support; 6) Haemodynamic instability (defined by mean systemic arterial pressure < 65 mmHg and/or diuresis < 0.5 ml/kg/h and/or patient non-responder to fluid challenge and/or amines support).

### Clinical recovery and transfer low dependency units (ward)

A patient is considered recovered when all the following criteria are fulfilled: 1) Apiretic patient for at least 72 h; 2) Respiratory rate < 22 breath/minute; 3) SpO2 in room air > 94% (or > 90% in case of chronic pulmonary disease); 4) Two negative samples (at least 24 h between the first sample and the second sample) for SARS-CoV-2.

Patients are considered eligible to be transferred to low-dependency units if all the following criteria are satisfied: 1) Apiretic for at least 24 h without use of anti-piretics drugs; 2) No respiratory support with CPAP, NIV or HFNC; 3) No new onset of complications or cardiovascular deterioration.

### Infection control

The rapid expansion of COVID-19 outbreak is indicative of efficient human-to-human transmission [83, 84]. Virus transmission via respiratory secretions in the form of droplets (> 5 μm) or aerosols (< 5 μm) appears to be the most common form of transmission [84, 85]. Healthcare workers are at high risk of contracting the infection particularly when applying respiratory devices such as oxygen cannulas or NIV, or when performing aerosol generating procedures on patients with COVID-19. Furthermore, the vulnerability in healthcare settings has been proved by the high numbers of reported cases of hospital-acquired infections [86]. Recent studies indicated that aerosol and fomite transmission of SARS-CoV-2 are plausible, as the virus can remain viable in aerosols for multiple hours and on surfaces up to days [87]. Therefore, hospitals present unique challenges during the process of protecting all healthcare providers from an infectious disease outbreak. Personal protective equipment (PPE) is an important component, but not the only one, of a system protecting healthcare providers from COVID-19 cross-infection. Indeed, appropriate use of PPE significantly reduces risk of viral transmission. Recommendations for the process of dressing and undressing for healthcare workers to avoid SARS-CoV-2 infection in COVID-19 unit and the safest use of “respiratory devices” in the COVID-19 unit are described in Table 5. Notably, after the adoption of a dedicated HDU for COVID-19 patients, none of the healthcare providers were infected by SARS-Cov-2. Healthcare providers are weekly screened with nasopharyngeal swab specimen to detect SARS-CoV-2 RNA from the upper respiratory tract. A serological test was performed for each healthcare provider after 3 months of COVID-19 HDU establishment. At the time of writing the present manuscript, only three pulmonologists and one nurse have been tested positive for IgG antibodies. However all of them developed the infection and they were tested positive by nasopharyngeal swab before the establishment of the COVID-19 HDU.

**Table 5:** Infection control procedures

***Dressing and undressing procedures for healthcare providers in COVID-19 high-dependency unit*** [34, 88, 89]***.***	
**Dressing**	**Undressing**
• Remove all personal items	• Remove the disposable gown and dispose of it
• Practice hand hygiene with soap and water or alcoholic solution	• Remove the first pair of gloves and dispose of them
• Wear a first pair of gloves	• Remove the glasses and sanitize them
• Wear the disposable gown over the uniform	• Practice hygiene of gloves with alcoholic solutions
• Wear FFP2/FFP3 covering the nose and mouth to minimize the space between the face and mask	• Remove FFP2/FFP3 by handling it from the rear and dispose of it in the container
• Wear protective full face shield or glasses	• Remove the second pair of gloves
• Put on a second pair of gloves	• Practice hand hygiene with alcoholic solutions or with soap and water
	• Shower including disinfection of the ears and mouth
***Protective equipment and recommendations for respiratory devices*** [34, 90, 91, 89, 92]	
**Procedures and/or device**	**Recommendation**
Aerosol-generating procedures (airway suction, bronchoscopy)	• Should be performed in negative pressure rooms • FFP3 mask for operator
NIV	• Use a double-limb circuit with a non-vented mask • Place three filters per ventilator: between the expiratory port and the ventilator; between the inspiratory port and the ventilator; near the patient’s mask • The interface with the lowest risk of aerosol emission is the helmet equipped with an inflatable neck cushion
Conventional oxygen therapy	• Do not use humidifiers • The patient has to wear the surgical mask to reduce contamination risk
HFNC	• The patient has to wear the surgical mask to reduce contamination risk • Nasal cannula must be completely inserted in the nostrils and secured with the elastic bands on the patient’s head to minimize lateral losses

*Abbreviations*: *NIV* Non-invasive ventilation, *HFNC* High-flow nasal cannula

## Conclusions

COVID-19 is a complex and heterogeneous disease. Several patients with COVID-19 disease show the signs and symptoms of a dis-regulated inflammatory response to infection leading to a systemic disease. Moreover, hypercoagulability seems an important problem leading to pulmonary embolism and DVT. Lack of experience in managing the disease and lack of highly effective antiviral drug are important limitations. Furthermore, there is need for improvement of ventilatory care. Increasing evidences have been showed the presence of biomarkers, such as IL-6 or ferritin, that can be targeted by off-label treatments. For these reasons a multidisciplinary approach, as the one that is used in our hospital seems to be reasonable. A multidisciplinary involvement of several figures is able to better identify treatable traits of COVID-19 disease, early identify patients who can quickly deteriorate, particularly patients with multiple comorbidities, and better manage complications related to off-label treatments. The management of patients in COVID-19 dedicated units is advisable for segregation purpose as well as for infection control. All the procedures adopted in our hospital cannot be generalized in other hospitals and different healthcare systems. However, we think that our experience and our point of view can be helpful for countries and hospitals that are now starting to face the COVID-19 outbreak.

## Supplementary information

**Additional file 1: Figure 1.** Map of Ca′ Granda Ospedale Maggiore Policlinico, Milan (Sacco, highlighted in red is the pavilion dedicated to COVID-19 HDU patients).

## Data Availability

Not applicable.

## Abbreviations

WHO: World Health Organization
SARS-CoV-2: Severe Acute Respiratory Syndrome coronavirus 2
HFNC: High Flow Nasal Cannula
NIV: Non Invasive Ventilation
IMV: Invasive Mechanical Ventilation
ARF: Acute Respiratory Failure
ICU: Intensive Care Unit
HDU: High Dependency Unit
SOP: Standard Operating Procedures
ER: Emergency Room
MDR: Multi Drug Resistant
CPAP: Continuous Positive Airway Pressure
DNR: Do Not Resuscitate
DNI: Do Not Intubate
RCT: Randomized Control Trial
CAP: Community Acquired Pneumonia
PEEP: Positive End Expiratory Pressure
ZEEP: Zero End Expiratory Pressure
BGA: Blood Gas Analysis
EKG: Electrocardiogram
PCT: Pro-calcitonin
CDC: Centers Disease Control
IDSA: Infectious Diseases Society of America
ARDS: Acute Respiratory Distress Syndrome
MRSA: Methicillin Resistant Staphilococcus Aureus
PE: Pulmonary Embolism
DVT: Deep Venous Thrombosis
DIC: Disseminated Intravascular Coagulation
ACE2: Angiotensin Converting Enzyme 2
RAS: Renin-Angiotensin System
ARB: Angiotensin Receptor Antagonists
PPE: Personal Protective Equipment
