# Supplementary material for: COVID-19 multidisciplinary high dependency unit: the Milan model
Source: Respir Res. 2020 Oct 9;21:260. doi: 10.1186/s12931-020-01516-8 (PMC7545383; doi:10.1186/s12931-020-01516-8)

Figure 1: Map of Ca' Granda Ospedale Maggiore Policlinico, Milan (Sacco, highlighted in red is the pavilion dedicated to COVID-19 HDU patients)

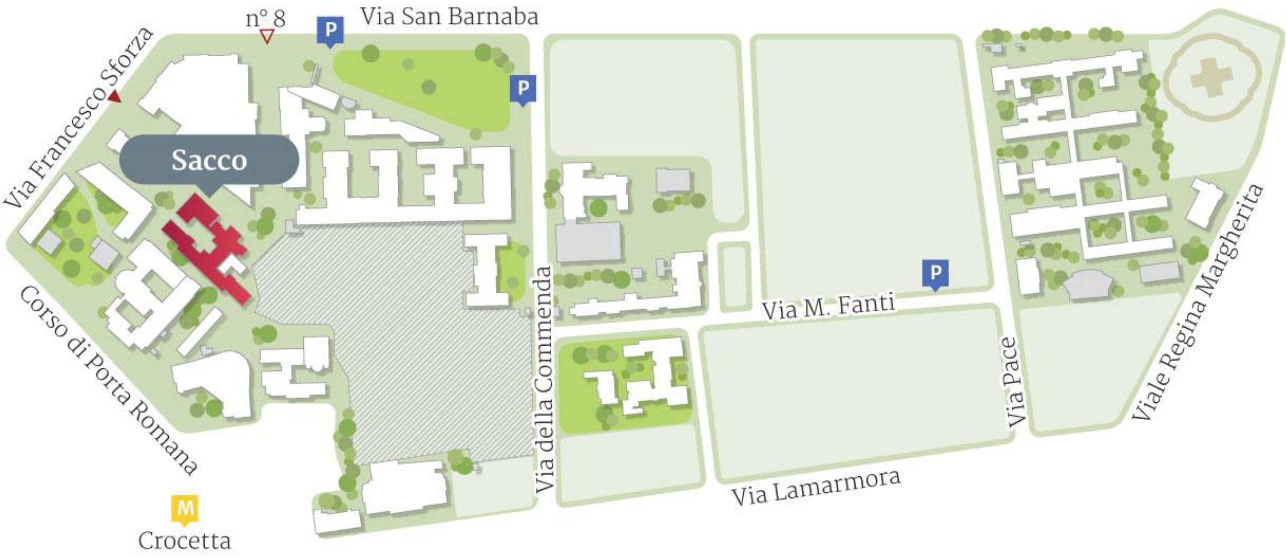

Supplement: Supplementary file 1 — Additional file 1: Figure 1. Map of Ca′ Granda Ospedale Maggiore Policlinico, Milan (Sacco, highlighted in red is the pavilion dedicated to COVID-19 HDU patients). [file 12931_2020_1516_MOESM1_ESM.pdf]
